# Soft tissue surgery as an initial treatment for hip displacement in spastic cerebral palsy

**DOI:** 10.1051/sicotj/2020036

**Published:** 2020-10-12

**Authors:** Luiz Antônio Angelo da Silva, Patricia Maria de Moraes Barros Fucs

**Affiliations:** 1 Santa Casa Medical School and Hospitals R. Dr. Cesário Mota Junior 61 01221-020 São Paulo/SP Brazil; 2 Department of Orthopaedic and Traumatology, Irmandade da Santa Casa de São Paulo, Santa Casa Medical School and Hospitals R. Dr. Cesário Mota Junior 61 01221-020 São Paulo/SP Brazil

**Keywords:** Cerebral pasly, Hip subluxation, Tenotomy, Muscle spasticity

## Abstract

*Objective*: To use the measurement of migration percentage (MP) to evaluate the long-term radiographic results of soft tissue surgery as the first treatment for hip displacement in spastic bilateral cerebral palsy. A secondary objective was to identify predictive factors of stability (i.e., less than 30% of MP in the long term), after surgical correction. *Methods*: In this longitudinal cohort study, we reviewed the electronic medical records and radiographs of all consecutive patients with cerebral palsy operated for the correction of hip displacement between 1984 and 2013 in a referral orthopedic public hospital in Brazil. Patients were included if they had received, as the first surgical procedure, soft-tissue release. All surgeries were bilateral and symmetrical. We used the available radiographs to evaluate the migration percentage (MP), acetabular index (AI), pelvic obliquity (PO) angle, head-shaft angle (HSA), congruence and femoral head sphericity, and function using the GMFCS (Gross Motor Function Classification System). *Results*: we included 93 patients, all operated before being 12 years old, with follow-up of 10 years in average, 73 (78%) of them with good results (MP < 30%). We found association between preoperative MP ≤ 40%, AI ≤ 25°, and postoperative symmetry with good results, with a cut-off value of 38% of MP and 27° of acetabular index being predictive. *Discussion*: The role of soft tissue releases remains controversial owing to small sample sizes, heterogeneity, variety range of ages, definitions used for outcome, and lack of statistical quality. Our results were better in combined tenotomies, in diparetic patients aged more than six years, and in patients with lower initial values of MP and AI. Radiographic variables had good correlation with each other and association with results, with cut-off values for MP and AI PRE.

## Introduction

The incidence of hip dislocation has been reported in 2.6–34% of children with cerebral palsy, resulting in pain and in quality-of-life reduction [1]. The basis for surgical treatment of hip dislocation in these cases is obtaining balance of the pelvis. However, there is still no consensus on which muscles should be addressed, the ideal age, the degree of dislocation that can be successfully treated [2], the post-operative care, the ways to evaluate the results and the factors that influence the results of soft tissue release.

Early detection of hip displacement is advocated, but it is not safe through clinical examination alone or by evaluating other risk factors [3]. It is necessary to perform radiographs. However, different limit values have been adopted as radiographic parameters in the literature, depending on the objective of the treatment and the type of evaluation. In addition, there are problems regarding accuracy, confidence, and inadequate patient positioning for radiographic assessments [2, 3]. The available studies are retrospective cohorts with patients of different age groups, radiographic and functional preoperative statuses and categories, length of follow-up, clinical subtypes, and evaluation criteria, leading to results that are difficult to compare [4]. Moreover, few studies have been published with a long follow-up time [5–7].

The main objective of this research is to use the migration percentage (MP) to evaluate the long-term radiographic results of soft tissue surgery as the first treatment for hip displacement in spastic bilateral cerebral palsy. The secondary objective is to identify predictive factors of stability (i.e., less than 30% of MP in the long term), after surgical correction.

## Methods

### Study design, setting, and ethics

This is a cross-sectional study, based on the review of electronic medical records and radiographs of all consecutive patients with cerebral palsy operated for the correction of hip displacement between 1984 and 2013 in a referral orthopedic public hospital in Brazil. This study was carried out between 2019 and 2020 after approval of the institutional review board (protocol 3.597.206). As the investigation was based on medical records review, no informed consent could be obtained specifically for this study, but all parents and caretakers consented for the medical procedures and were informed about the possibility of use of aggregated data from anonymized records.

### Participants

We reviewed the hospital electronic medical records in search of cases of children with spastic bilateral cerebral palsy who received, as the first surgical procedure, soft-tissue release. We used the following inclusion criteria:

- age of 12 years or less at the first surgery;
- available preoperative and postoperative radiographs, with anterior-posterior view of the hip;
- at least one hip with migration percentage (MP) of less than 90%;
- data in the medical record comprising at least four years of follow-up after surgery.

All surgeries included in this study were bilateral, as per the departmental routine, and symmetrical. They were conducted starting with a medial incision over the long adductor muscle to access the set of adductors, indicated by the clinical test showing the abduction limitation <30°. The most proximal tenotomy possible was performed by the same route in case of hip flexion contracture >30°, and hamstrings were released distally in case of shortening on physical examination.

### Variables, measurements and bias reduction

We used the available radiographs to evaluate the migration percentage (MP) and also to perform the following set of additional measurements:

- Acetabular index (AI);
- Pelvic obliquity (PO) angle;
- Head-shaft angle (HSA).

For these measurements, we used the ImageJ processor (National Institutes of Health) over the digital radiographs.

To avoid bias, the same evaluator took these measurements twice (20), aiming to get the same result. If there was difficulty in choosing reference points in the images for the lines drawing, some measurements were not taken. We could not evaluate AI in 6% of the images, where there was extreme pelvis rotation. We could not evaluate HAS in cases of great exposure of the small trochanter or when the femur had a “scoliotic” aspect.

**Figure 1:**
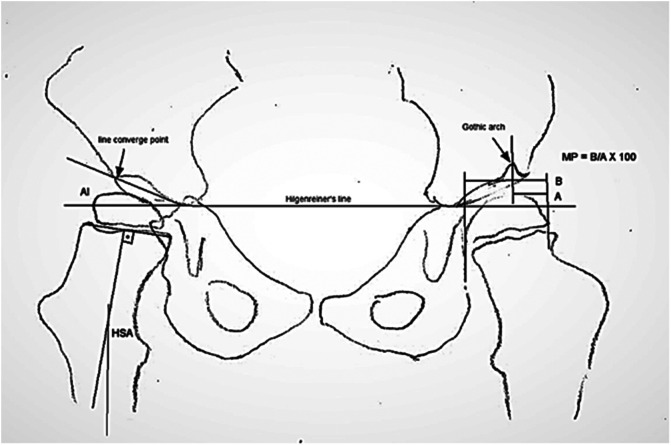
References for the calculation of migration percentage (MP) in the presence of a Gothic arch, acetabular index (AI) in the case of oval image, and the head-shaft angle (HSA).

In the presence of a Gothic arch, we drew a Perkins’ line at the apex of the arch; if there was pelvic anteversion, we used a bisector of the oval image by double acetabular lines (Figure 1). We did not measure AI or HSA in cases with extreme rotations, because it could compromise accuracy.

We verified in the radiographs if the postoperative MP was less than 30%. In these cases, and if the patient was not submitted to osteotomy as a revision surgery, the result was registered as good. If the patient underwent soft tissue release, but MP was still less than 30%, the result was also good.

We also evaluated, in the postoperative radiographs, the morphology of the hips for congruence and femoral head sphericity.

Besides demographic data, we collected the functional evaluations from the medical records, which were performed at the time using the GMFCS (Gross Motor Function Classification System).

### Statistical analysis and study size

This study used a convenience sample comprising all medical records available since inception of the electronic medical records in the hospital, with no previous sample size calculation.

We described data as frequencies and averages. We used chi-squared or Fisher exact test as appropriate for qualitative and Student’s *t* test or Mann–Whitney for quantitative variables to examine the association between them and good results. We calculated the correlation between quantitative variables using scatter plots and Pearson’s or Spearman’s correlation analysis. We used Epi-Info 7.2 and SPSS 13.0 for receiver operator characteristic (ROC) curves. We adopted *p*-values < 0.05 as significant.

## Results

In the study period, 93 patients were operated and included in the study, 57% boys. They had all been operated before being 12 years old, in average at 6.8 years (standard deviation [SD] = 3 years; range: 2.4–12 years). The mean follow-up period was 10.3 years (SD = 6) years, most (73%) with follow-up longer than 6 years, and 54% reaching skeletal maturity at the last follow-up. The age at the final evaluation was in average 17 years. All cases had digital radiographs available for the PRE and final evaluations.

Among all patients, 73 (78%) were considered to have good results. For 63 patients (68%) with subluxated hips initially (MP > 25%), the result was considered good. For all the 30 patients with hips “at risk”, the result (MP of 25% or less) was good. There was no significant difference of age between patients considered as having good or bad results. We found MP PRE > 40% associated with the age of 4 years and MP PRE ≤ 40% aged 7 years with statistical significance.

Most patients were diparetic (65%), and 90% of these cases were associated with good results (*p* < 0.001). The functional level was available for 80 patients, showing 65% of them with GMFCS (Gross Motor Function Classification System) IV–V (*p* = 0.72). GMFCS remained unchanged at 59%, 25% had functional worsening, and 19% of patients had improvement in the follow-up (100% associated with good results; *p* = 0.03).

Combined tenotomies (70%) had better results than the isolated ones (*p* = 0.03) especially those involving the adductor, psoas, and hamstrings (16%; *p* = 0.02), or when they were combined with hamstring tenotomies (46%) *p* = 0.007.

**Figure 2:**
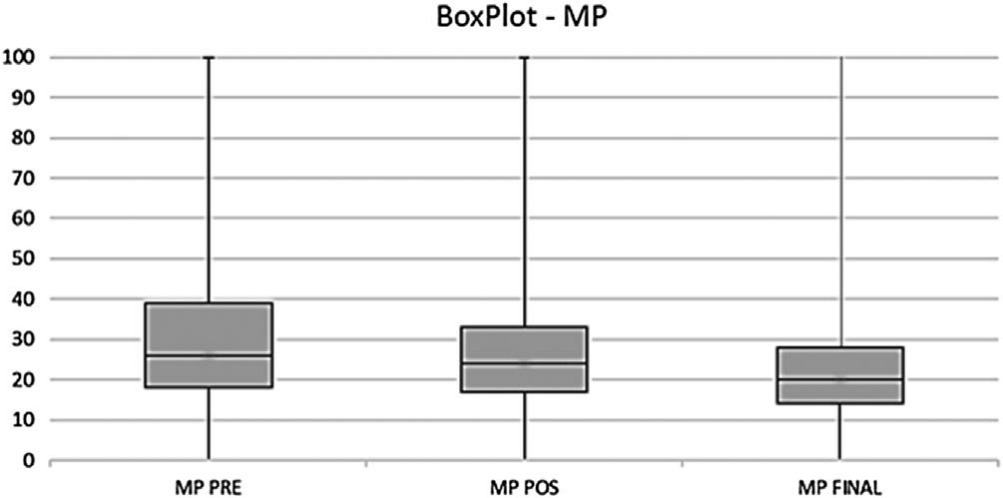
Preoperative (PRE), postoperative (POS), and final medians of migration percentage (MP): 26%, 24% and 20% respectively.

We evaluated the association of the mean MP, AI, HSA, PO measurements with the good or bad results (Table 1). We also evaluated the difference of MP between hips preoperatively and the association with good or bad results: preoperatively 8 and 17, respectively (*p* = 0.02); postoperative 7 and 20, (*p* = 0.15); and final 5 and 21 (*p* < 0.001). The MP mean measurements (Figure 2) decreased from the preoperative to postoperative and final follow-up evaluations (30%, 27% and 22% respectively), and the same happened with AI (25°, 24° and 23°) and HAS (155°, 154° and 148°).

**Table 1:** Preoperative (PRE), postoperative (POST) and final mean radiographical measurements and differences according to Student’s *t* or Mann–Whitney’s tests.

Variables	PRE	*p*	POST	*p*	Final	*p*
MP (Good)	25%	<0.001	20%	<0.001	17%	<0.001
MP (Bad)	51%		49%		44%	
AI (Good)	24°	<0.001	21°	<0.001	21°	<0.001
AI (Bad)	31°		34°		31°	
HSA (Good)	154°	0.35	153°	0.10	151°	0.09
HSA (Bad)	156°		158°		144°	
OP (Good)	2°	0.28	3°	0.04	3°	<0.001
OP (Bad)	3°		5°		8°	

MP = migration percentage; AI = acetabular index; HSA = head-shaft angle; PO = pelvic obliquity angle.

**Figure 3:**
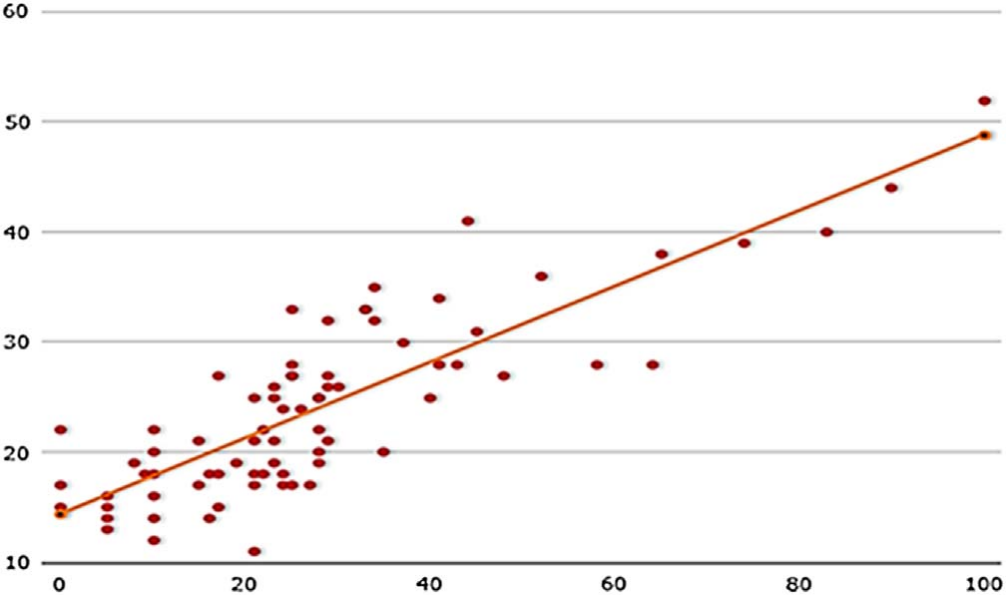
Distribution of migration percentage (MP) and acetabular index (AI) after surgery.

Scatter plot shows strong positive correlation between MP and AI postoperatively (Figure 3), with 0.832 according to Spearman’s linear regression, and final (0.786, Pearson). It shows also moderate correlations between MP and AI preoperatively (0.624, Pearson), and MP and HSA (0.535, Spearman), AI and HSA (0.495, Spearman) and MP and PO (0.475, Spearman) postoperatively.

Among all patients, 41% needed revision surgeries for bone or soft tissue. These had higher MP mean (36%) compared with children not reoperated (27%; *p* = 0.01). These cases were more frequently isolated adductor releases (*p* = 0.005).

Additional soft tissue was necessary for 32% of the patients, considered as adjustments. Bone corrections were performed in 10 of the 93 patients, for the hip with worst results: varus derotation osteotomy [2], pelvic and femoral osteotomy [8], salvage [1], bilateral [9]. The MP mean preoperatively was always 52% versus 28% for patients undergoing or not the bone revision surgery (*p* < 0.001).

The morphology evaluation showed the following results for the worst hip of each patient: 81 were spherical and congruent, 2 spherical and incongruent, and 2 needed salvage. Among the 93 patients, 89 (96%) had final congruency.

## Discussion

The studies about spastic cerebral palsy in the literature tend to be based on heterogeneous groups, which makes their comparison and understanding difficult [10, 11]. The inclusion criteria adopted in our study provided a homogeneous sample of children of the same age range, all with spastic bilateral cerebral palsy, and submitted to soft tissue surgery as the first procedure. This study showed that diparetic children (*p* < 0.001) and those operated after six years of age (*p* = 0.02) had better results, in contrast to findings in the literature with younger children [8]. Our study thus suggests that it is worthwhile to start the treatment with conservative approaches in this range age.

Adductor tenotomies, and surgeries involving the psoas and hamstrings (16%), or those that associate hamstrings had better results compared to adductor surgery alone or with psoas, with statistical significance. Kalen and Bleck [1], found better results including the psoas than with isolated adductor tenotomies, and other authors include the psoas release frequently [6, 12].

We observed a migration of patients with GMFCS levels I–III to the IV–V level during follow-up, showing functional worsening even with the treatment, but most patients’ function remain unchanged (59%). Predominant preoperative levels were IV–V (65%).

According to other authors [6, 13, 14] view, a second soft tissue surgery (that happened in 32% of the patients) was not considered a complication, and we did not exclude these patients from the analysis. Bone surgeries (a poor outcome criterion) were performed in the follow-up for ten patients. Revision surgeries (both for soft tissue or bone correction) were registered for 41 patients, and associated with higher MP preoperatively (36%; *p* = 0.01) and with isolated adductors release, more than with combined surgeries (*p* = 0.005). In fact, other studies have evidenced the need for additional surgeries in 20–60% [4, 5, 14].

As already mentioned, we could not evaluate AI in 6% of the images, where there was extreme pelvis rotation, and HSA in cases of great exposure of the small trochanter or when the femur had a “scoliotic” aspect, as argued for by Tönnis [15]. However, this decision is unusual in other studies, where positioning and contractures have a great influence on measurements and results [9].

MP preoperative values were between 0% and 89%, mostly below 39% (Figure 2). We found evidence between age of 4 years old and MP > 40%, in the same way 7 years old and MP ≤ 40% (*p* < 0.001), showing a relationship between age and MP value in surgical indications similar to the indication profile of Presedo et al. [6].

Preoperative 25% and 51% MP mean were associated with good and bad results, respectively (*p* < 0.001), with average decreasing with treatment until final evaluation. Shore et al. [7] found preoperative MP averages of 21% and 45%, and Turker and Lee [5] found 33% and 43% associated with success and failure, showing that the initial degree of subluxation can interfere with soft tissue surgery success [5, 7, 16].

Mean AI decreased one point preoperatively, postoperatively and in the final evaluation (25–24–23 degrees), although other studies have not found improvement in AI after soft tissue procedures [17, 18]. Preoperative AI ≤ 25% had 96% of hips associated with good, and preoperative averages 24° versus 31° associated with good and bad results, respectively (*p* < 0.001).

We evaluated the symmetry principle, based on the difference between MP values of the hips. Macias-Merlo et al. [19] found differences in the treated and control groups 10% and 35%, respectively, with statistical significance. We infer that MP differences ≤10% between hips give rise to symmetrical hips, and larger differences to asymmetric hips. The exploration of the concept showed a linear improvement of the MP differences toward symmetry. Postoperative symmetry was associated with the results (*p* = 0.003).

**Figure 4:**
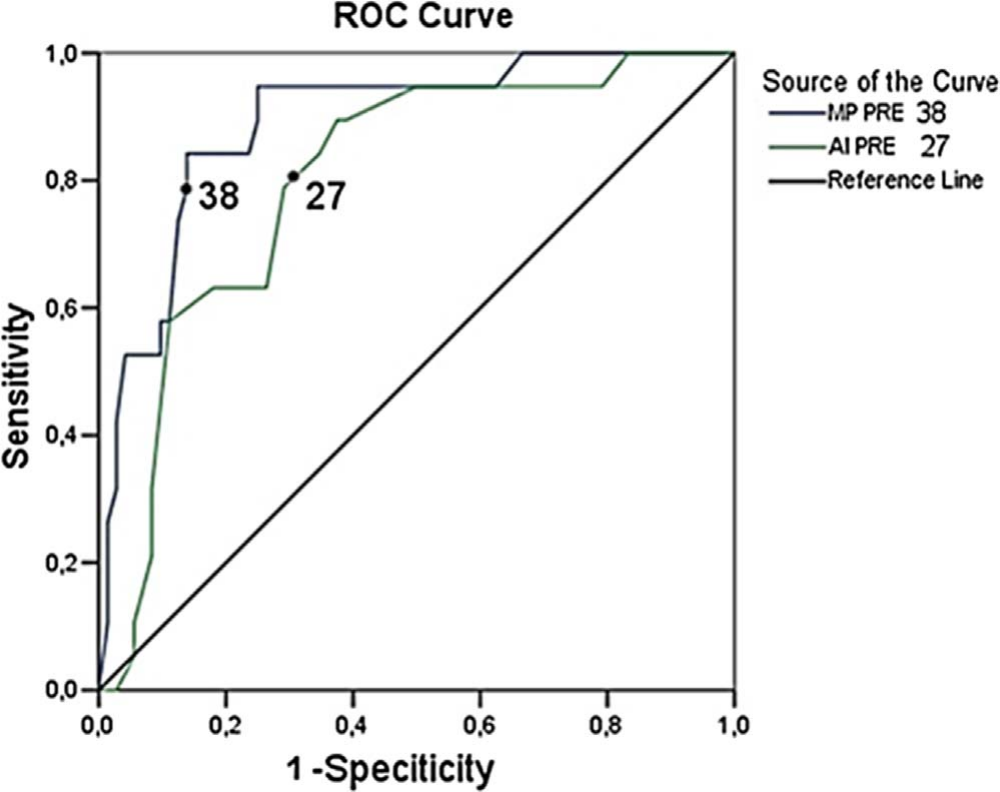
Receiver operator characteristic (ROC) curve with cut-off values of 38% (AUC 0.897) for migration percentage (MP), and 27° (AUC 0.769) for acetabular index (AI), before (PRE) surgery.

The radiographic parameters had strong and moderate correlations with each other, and the ROC curves showed excellent preoperative cut-off values for MP 38% and AI 27° (Figure 4). Cornell et al. [2] found MP ≥ 40% and AI ≥ 27°, responding less to treatment [4].

Pelvic obliquity is often associated with bilateral cerebral palsy, influencing outcomes [1, 8]. Averages of 3° and 5° postoperative were associated with good and bad results (*p* = 0.04), respectively in our study. Postoperative measurements were associated with the result, with statistical significance (Table 1).

We consider MP ≥ 30% as a clear abnormal result, that shows the subluxation is subject to progression in the child with severe involvement [11, 20]. The overall result was good for 73 of 93 of our patients (78%) comparable to that of another study using similar methodology [6].

For 63 (68%) patients with initial subluxated hips (MP > 25%) it was good, and for 30 patients with “hips at risk” (MP ≤ 25%) it was 100% good shows the value of the initial MP as a predictive factor. Bowen and Kehl [12] consider it necessary to identify precisely which hips will subluxate without treatment (studies show 26–78%), however, without more complete knowledge of the natural history of each hip, or the impossibility of close monitoring, most surgeons will intervene in smaller MPs, as in our study patients.

In the literature, success rates have been related to the duration of the follow-up, and high rates (70%) are found in follow-ups of less than 5 years [4, 8]. Turker and Lee [5] claim that a follow-up of more than six years is necessary to determine the outcome of soft tissue surgery. A study found 73% of patients with a follow-up longer than six years and, although not all patients reached skeletal maturity, 89% were more than 10 years old in the final evaluation, older than the age when the highest incidence of dislocation occurs, although some patients suffer from luxation during the growth spurt [11]. Hips that are morphologically classified as 96% congruent at the final evaluation suggest that the patient will probably have a good evolution and favorable anatomy in future procedures that preserve the joint (tenotomies or reconstructive surgeries).

## Conclusions

This study has shown that 78% of children with cerebral palsy and spastic hips have good results, evaluated by migration percentage in radiographs, after soft tissue release, not needing revision bone surgeries in the follow-up. Preoperative migration percentage of less than 40% and acetabular index ≤ 25°, and postoperative symmetry were significantly associated with good results, with a cut-off value of 38% of migration percentage and 27° of acetabular index being predictive. The results were better in combined tenotomies, in diparetic patients more than six years old, and in patients with lower initial values of migration percentage and acetabular index.

## Conflicts of interest

All authors confirm that they did not receive any internal or external fund for the presented work. All authors have no conflicts of interest to declare.

## Funding

No funding was received for the submitted work.
